# Assessing transferred energy in drone impacts using rigid impactor tests

**DOI:** 10.1371/journal.pone.0342560

**Published:** 2026-02-12

**Authors:** Zdeněk Svatý, Pavel Vrtal, Luboš Nouzovský, Petr Zlámal, Jakub Nováček

**Affiliations:** 1 Department of Forensic Experts in Transportation, Faculty of Transportation Sciences, Czech Technical University in Prague, Prague, Czech Republic; 2 Department of Forensic Experts in Transportation, Faculty of Transportation Sciences, Czech Technical University in Prague, Prague, Czech Republic; 3 Department of Forensic Experts in Transportation, Faculty of Transportation Sciences, Czech Technical University in Prague, Prague, Czech Republic; 4 Department of Mechanics and Materials, Faculty of Transportation Sciences, Czech Technical University in Prague, Prague, Czech Republic; 5 Department of Forensic Experts in Transportation, Faculty of Transportation Sciences, Czech Technical University in Prague, Prague, Czech Republic; Virginia Tech, UNITED STATES OF AMERICA

## Abstract

The rapid development of unmanned aerial systems (UAS) has led to their widespread use and affordability. A major safety issue is understanding the health consequences of drone-human collisions, which largely depends on rigorous testing methods. The aim of this paper is to determine the response of the anthropomorphic test device (ATD) using a rigid impactor with gradually increasing impact kinetic energy (KE) and to obtain a validated dataset for subsequent comparisons in case of vertical impact to the top of the head. A total of 30 tests were performed with kinetic energy at impact ranging from 5 to 180 J. It was assumed that the majority of the kinetic energy during impacts was absorbed by the ATD head. This enables the derivation of scalable results across various impact energies and orientations. These results were subsequently compared with actual UAS impact tests. The findings suggest that with increasing kinetic energy of the UAS impact, the percentage of transferred energy decreases. For this reason, it is not possible to confirm a linear trend similar to that observed in tests with rigid impactors. This phenomenon is particularly evident in the design of the DJI Phantom, whose plastic frame underwent significant deformation, thereby reducing energy transmission to the head. The comparison with impactor tests showed that, although the UAS was potentially able to reach a KE of up to 280 J at a limit speed of 20 m/s, the transmitted energy was only about 20%. This observed trend was confirmed by biomechanical criteria, including Peak Head Acceleration, Head Injury Criterion, 15 ms (HIC15), and Neck Injury Criterion (Nij). In cases where testing facility capabilities are limited and tests cannot be performed at the critical speed or terminal velocity, the HIC15 relationship to KE seems to be more accurate than peak head acceleration. It should be noted that the proposed approach is validated only for vertical, top-of-head impacts. For these reasons, the safety criteria currently used for UAS, which are primarily based on a linear energy transfer assumption, may lead to overly restrictive injury predictions when applied to the more pliable and deformable UAS structures.

## Introduction

The rapid development of unmanned aircraft systems (UASs) and related technologies in recent years has led to their widespread expansion worldwide. The increasing affordability and availability of these systems have enabled their exponential growth and use, even for the general public. However, the implementation of new regulations for UAS has proven to be a global challenge for aviation authorities, who have traditionally focused on safety related to traditional air traffic and its passengers.

A major part connected with safety is to determine in what cases a drone-human collision will have health consequences. How the testing is conducted plays a significant role in assessing this issue. Currently, the computation simulations represent the most suitable solution for rapid validation and safety assessment. The option to easily change the tested scenarios, observe the effects of probable impact configuration or easily repeat or validate various measurements. However, the accuracy and validity of such simulations are inherently dependent on the used models, thus, available information about the UAS and its characteristics and their validation against performed crash tests. In context to this, considerations to the limitations, correct usage and understanding of validation datasets is crucial.

In recent years, new standardized procedures were introduced and enabled the implementation of crash testing as an option for safety assessment. But even here lie several issues. One of them is for example the limitation of defined usage of the 50th percentile male Hybrid III that was developed in 1974 [1–3].

The ASTM standard [4] enables utilization of simplified anthropomorphic test device (ATD) consisting of a head and neck, which was proposed in the Assure research [5,6]. The simplified ATD shows a significantly different response (up to 1,5 higher head acceleration) in comparison with full scale-ATD [5]. Utilization of this approach may lead to a significant decrease of the corresponding costs of the testing, but also may lead to over-restrictiveness and limit the compatibility between acquired data.

However, the main issue is that most conclusions, proposed requirements, and test procedures rely almost exclusively on the ASSURE research [5,6]. Although the contribution of Assure is unquestionable, the test results were never validated by other researchers or testing facilities. The corresponding costs, time demands, equipment and necessary knowledge present a significant limitation that limits the possibility of the verification.

Following the findings, the authors of this paper performed 49 new drop tests with different UAS (up to 250g). These tests were presented in a previous publication [7,8]. The tests were carried out with different drones, impact speeds and different impact configurations of UAS (horizontal position, upside down, angled). The drop height differed based on the target impact velocity or impact KE. The aim was to acquire data for assessment of the repeatability of tests, validation data against already performed tests and also to compare different types of UAS under similar conditions, such as similar impact speed or KE. Together with these tests, a comprehensive database was created containing information with a total of 184 drop tests performed also in other research projects [9].

Various test series showed that lightweight UASs under 250 g do not lead to a high head or upper neck reaction [7]. These tend to have lower terminal velocities, and even in cases where the terminal velocity is higher or not predicted correctly, it does not change this conclusion. Furthermore, the material of the frame for these small drones does not seem, based on the performed tests, to significantly affect the resulting consequences.

The available data sets from previous studies [5] and the tests performed by the authors [7] show the limitations of the prediction capability related to the kinetic energy (KE) at impact for the deformable and fragile UAS structure. The kinetic energy calculations are only dependent on mass and velocity, the main study presented by Feinstein et al [10] or by, e.g., Jansen et al [11].

Table 1 shows the correlation (R^2^) for 184 tests from the previous mentioned database [9]. While there is a high correlation between groups based on automotive models, the KE are predicting higher risks than automotive human vulnerability models (HVMs). Thus, their mutual correlation is rather low.

**Table 1:** Correlation between the HVMs for crash tests in the database (184 tests).

*Correlation of HVMs for crash tests from the database (184 tests)*	*PoF RCC 321 Head (Feinstein)*	*PoF Janser Head*	*Skull fracture AIS2 (Peak head acceleration Mertz)*	*Skull fracture AIS2 (HIC15 Mertz)*	*Skull fracture AIS2 (HIC36 NHTSA)*	*Head AIS 3 (HIC15 Prasad)*	*Head AIS 3 (HIC15 NHTSA)*	*Brain Injury AIS4 (HIC15 Prasad)*	*Brain Injury AIS4 (HIC15 Mertz)*	*AIS 3 injury (Nij)*
**PoF RCC 321 Head (Feinstein)**	**1.00**									
**PoF Janser Head**	**0.95**	**1.00**								
**Skull fracture AIS2 (Peak head acceleration Mertz)**	**0.36**	**0.37**	**1.00**							
**Skull fracture AIS2 (HIC15 Mertz)**	**0.23**	**0.25**	**0.76**	**1.00**						
**Skull fracture AIS2 (HIC36 NHTSA)**	**0.34**	**0.37**	**0.89**	**0.94**	**1.00**					
**Head AIS 3 (HIC15 Prasad)**	**0.36**	**0.39**	**0.92**	**0.85**	**0.97**	**1.00**				
**Head AIS 3 (HIC15 NHTSA)**	**0.27**	**0.29**	**0.79**	**0.99**	**0.96**	**0.88**	**1.00**			
**Brain Injury AIS4 (HIC15 Prasad)**	**0.27**	**0.29**	**0.84**	**0.96**	**0.98**	**0.94**	**0.96**	**1.00**		
**Brain Injury AIS4 (HIC15 Mertz)**	**0.23**	**0.24**	**0.76**	**1.00**	**0.94**	**0.85**	**0.99**	**0.96**	**1.00**	
**AIS 3 injury (Nij)**	**0.39**	**0.39**	**0.66**	**0.77**	**0.77**	**0.67**	**0.81**	**0.71**	**0.76**	**1.00**

Following the above conclusions, the most probable explanation of the discrepancy between the kinetic energy vulnerability models and other criteria can be identified in the amount of transmitted energy from the UAS to the head of the ATD. Due to the design and material characteristics of the UAS, a significant part of the kinetic energy is transferred into deformations and movement of individual parts of the structure. Unfortunately, due to the complexity of the movement of individual components and the absence of accurate material characteristics of the UAS structure, the amount of transferred energy cannot be easily determined even if the crash tests are utilised. Nevertheless, it is the transferred energy to the affected part or limb that determines the rate of subsequent injury.

The aim of this paper is to determine the response of the anthropomorphic test device using a rigid impactor with gradually increasing impact kinetic energy and to obtain a validated dataset for subsequent comparisons. It is assumed that the information obtained from the impactor tests will help to further define the vulnerability criteria for the evaluation of specific drone impact tests. The assumption is that in impactor tests, most of the kinetic energy at impact is transferred to the ATD head. As a consequence, scalable results can be obtained for different KEs and impact orientations. The results obtained will then help to determine whether the transferred energy assumption is set correctly or not.

### Current legislation

The UAS regulation in the EU is currently in a transitional phase. The rules and regulations for the operation of the UASs with a mass under 150 kg used to be defined by the individual member states and varied widely. The regulation (EU) 2019/945, 2019/947 [12,13], later amended by several Commission Implementing Regulations over the years, unified the rules for UAS across the member states. The regulation introduced categories and subcategories based on the corresponding risk levels and defined operational limitations and requirements. With regard to the focus of this study, main point is the definition of drone classes (C0 – C6). Considering the ground risks, it contains mass thresholds of 250 g and 900 g, a maximum operating speed of 19 m/s, a maximum flight altitude of 120 m and a maximum energy that is transferred to a human head during impact (80 J). The values are based on Opinion No. 01/2018 [14], which summarised the previous conclusions under the Rulemaking task RMT.0230 [15,16]. The main parameters, relevant to the impact assessment, are the relations to the set threshold values. The Class C0 drones are limited by the 250 g mass threshold, limited speed of 19 m/s and maximum attainable height above the take-off point limited to 120m. Class C1 drones are defined with mass limit of 900 g or the maximum energy transmitted to the human head of 80 J [14].

The experts under the Aerospace and Defence Industries Association of Europe — Standardization (ASD-STAN) have prepared the prEN4709 standards [17] to provide technical specification and verification methods to support compliance with regulations.

The UASs in the USA are regulated by the Code of Federal Regulations (CFR) 14 part 107 [18]. The limits for operation are an altitude of 122 m, with a maximum speed of 44 m/s and an upper weight limit of 25 kg. Subsequent rule by the Federal Aviation Administration (FAA) [19] defined three categories of permissible operations over people based on the risk of injury. Considering the ground risks, it contains the mass threshold of 250 g and two energy thresholds of 14.9 J (11 ft-lbf) and 33.9 J (25 ft-lbf). The categories were later supplemented with the fourth category, which is based on the UAS having an airworthiness certificate. This was based on public demand and also on the research performed by Assure. The regulation is subsequently supplemented by several ASTM standards (American Society for Testing and Materials) which define among other also the design, construction, and test requirements for a UAS, or assess the safety of UASs, such as ASTM F3341/F3341M [4].

The 250 g presents an already used limit for the registration of UASs. The mass threshold was derived with a standard aviation risk assessment formula used in consideration of manned aircraft safety [20]. An acceptable risk level was achieved with a series of assumptions to estimate the probability of a lethal event occurring per a UAS flight hour. The energy threshold limit of 80 J served as the basis [21]. Using this energy equates to an object weighing 250 g traveling at a terminal velocity of 25 m/s.

In ASTM F3389/F3389-21 in Method D, it is possible to further encounter specific parameterization of tests. Tests with the rigid impactor should be conducted at the KE that is equivalent to the CAA-defined thresholds (14,9 J or 33,9 J/ 11 ft-lbf or 25 ft-lbf). The impactor weight is limited to a range between 0,45–2,27 kg (1–5 lb) and an approximate frontal area of 38–77,42 cm^2^ in a symmetrical fashion (6–12 in^2^) [4].

Subsequently, the results between the impactor and UASs test are compared through the determined peak head acceleration, HIC15, Nij and upper neck compression (vertical impacts). If the impact induced values are higher than those for UASs, the tests are considered as a pass.

The European standard (prEN 4709 part 1) [17] also allows a similar procedure, but using simulation (finite element analysis) or statistical methods. However, no mention is made of practical testing.

As a result, no other comparable standards or regulatory frameworks addressing human vulnerability or impact risks were identified outside the EU and the US.

## Materials and methods

**Fig 1:**
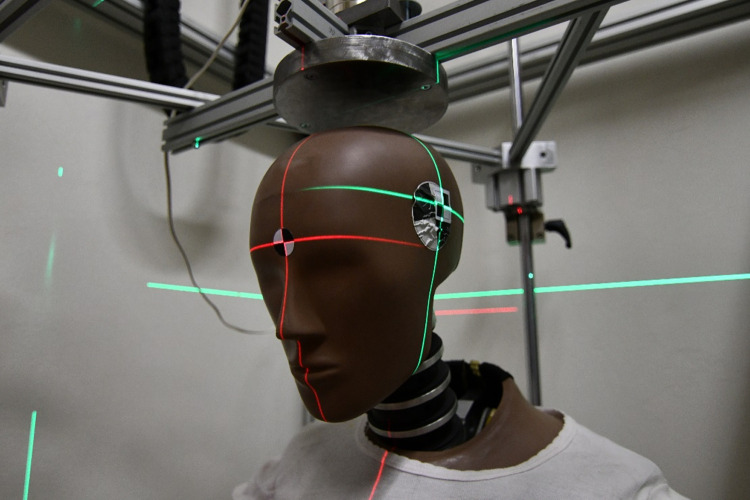
Rigid impactor testing setup.

The general idea of the tests was to determine the response of the ATD to a gradually increasing impact KE of a rigid impactor and to obtain a validated dataset for subsequent comparison. However, the impactor testing had to implement a certain level of simplifications and limitations. Due to the previous focus of the crash tests, it was decided that the proposed solution will be tested only for a vertical impact to the top of the head of ATD and other impact orientations will not be considered (Fig. 1).

A drop machine for the passive safety crashworthiness of cyclist helmets was modified and utilized for testing. A steel impactor with a special mount was designed and machined out of an aluminum block in order to simulate a very rigid body that could only move in the Z axis and had limited options for rotation. The diameter of the impactor was selected to be larger than the potential contact area on top of the head of a 50th percentile male Hybrid III [1], and it was approximately 200 cm^2^. Also, the scalability of the drop tower was considered and an option for increasing or decreasing the impactor mass was implemented. The height of the impactor above the head of the ATD was determined through a laser distance sensor with an accuracy of 0.1 mm.

The ATD was fitted with high speed Kistler instrumentation, with 27 channels measured, including head, chest, and pelvis accelerometers with a range of 1,500 g; measurement of force and torque in the upper and lower parts of the neck; chest compression, lumbar force and torque, and deflection of the knee. The weight of the dummy was 77.7 kg and the sitting height was 884 mm. The ATD was placed on a specially created chair made of stiff and rigid material to prevent any negative effects on the measurements. The posture of the ATD was set identically as during the crash tests, with the head centre of gravity (CG) in the middle of the impact zone and directly above the lower neck. Detailed information on the sampling rates and sensor models for all measurement channels is provided in the accompanying data repository.

The tests were captured with three high speed cameras. The first camera captured the front side of the impact with a frame rate of 2000 fr/s and was backed by a second camera with a frame rate of 1000 fr/s. The third camera captured the right side with a frame rate of 1000 fr/s. The impact velocity was determined from the recordings of cameras one and three, along with a laser time-gate.

A total of 30 tests were performed with kinetic energy at impact ranging from 5 to 180 J. Due to the limited height of the drop facility, the weight of the imapactor was increased from 9.07 kg to 12.78 kg by fixed mounting of additional weights to the top of the impactor for KE above 100 J. Other test parameters were not changed. To validate the variance of the measured data, the tests at 50 J and 100 J were performed five times for the lower weight impacts, and two times for the heavier configuration tests at 50 J and 100 J in order to enable subsequent estimations of the effects of increased mass.

### Rigid impactor tests results

The following Table 2 and Table 3 summarize the results of tests performed with a rigid impactor. Table 2 lists the main parameters of the impact and the resulting response from the ATD head and upper neck (peak head acceleration, 3 ms head acceleration, HIC15, Nij, and upper neck forces and moments). The values of the monitored parameters were measured by sensors incorporated in the ATD head and upper neck. These values were then evaluated and processed to serve as input values for the evaluation of the HVM criteria. Depending on the requirements of the input pre-processing of the variables for the individual criteria, the variables were properly processed (e.g., filtering with the CFC1000 filter for the HIC15 criterion). Accelerations, forces, torques were each filtered with CFC filters in accordance with FMVSS regulation [22]. No other mathematical procedures or adjustments of the data were applied. The measured values which reach the threshold limit values are highlighted in red, and the remaining values are indicated in relation to the threshold values. It should be noted that a combined measurement uncertainty was determined for the measured data. Data from impactor No. 18 (Impact KE = 79.46 J) can be given as a model example. At a speed of 4.19 m/s, the combined expanded uncertainty is ± 0.17 m/s, which corresponds to an approximate 4% error in speed measurement.

**Table 2:** Rigid impactor tests results – measured values.

Internal ID	Impactor material	Impactor mass	Height	Impact velocity	Impact KE	Maximum Resultant Head Acceleration	Head Accel. (3ms)	Head injury criterion (HIC15)	Neck injury criterion (Nij)	Upper neck tension (Fz)	Upper neck compression (-Fz)	Upper neck flexion (-My)	Upper neck extension (My)	Upper neck shear
Unit	–	[kg]	[m]	[m/s]	J	(g)	(g)	–	–	N	N	Nm	Nm	N
Limit value	–	–	–	–	80	200	80	700	1	6806	6160	310	135	3100
**Impactor_12**	**Steel**	9.07	0.06	1.08	5.32	23.31	7.48	4	0.28	14	1266	7	20	183
**Impactor_11**	**Steel**	9.07	0.121	1.51	10.29	45.57	11.42	13	0.37	6	1763	12	25	272
**Impactor_13**	**Steel**	9.07	0.241	2.12	20.38	81.12	14.77	46	0.48	29	2404	18	33	401
**Impactor_14**	**Steel**	9.07	0.36	2.58	30.28	113.53	18.97	102	0.57	37	2853	21	37	505
**Impactor_15**	**Steel**	9.07	0.483	3.00	40.71	155.90	22.17	175	0.63	39	3180	25	40	583
**Impactor_01**	**Steel**	9.07	0.563	3.25	47.84	157.08	21.17	202	0.69	32	3447	29	49	635
**Impactor_02**	**Steel**	9.07	0.591	3.30	49.39	181.01	25.74	248	0.70	48	3511	31	48	652
**Impactor_22**	**Steel**	12.78	0.409	2.79	49.56	163.68	22.90	214	0.73	45	3671	12	48	656
**Impactor_04**	**Steel**	9.07	0.599	3.31	49.66	186.09	27.30	253	0.70	24	3470	31	49	646
**Impactor_03**	**Steel**	9.07	0.598	3.32	49.90	182.35	26.26	245	0.69	108	3454	30	47	643
**Impactor_05**	**Steel**	9.07	0.6	3.32	50.08	189.05	26.88	254	0.70	25	3456	31	48	654
**Impactor_23**	**Steel**	12.78	0.413	2.80	50.10	168.18	24.54	232	0.74	30	3749	13	47	670
**Impactor_16**	**Steel**	9.07	0.721	3.65	60.38	238.12	34.06	392	0.75	26	3754	30	47	723
**Impactor_24**	**Steel**	12.78	0.571	3.30	69.59	236.19	30.85	453	0.85	28	4299	20	53	792
**Impactor_17**	**Steel**	9.07	0.842	3.92	69.69	276.85	35.68	532	0.80	27	3978	32	50	781
**Impactor_18**	**Steel**	9.07	0.96	4.19	79.46	318.53	37.70	699	0.85	28	4229	34	52	840
**Impactor_19**	**Steel**	9.07	1.081	4.42	88.60	355.73	36.13	889	0.89	102	4436	36	54	908
**Impactor_28**	**Steel**	12.78	0.819	3.90	97.19	359.62	48.41	995	0.98	64	4986	28	60	975
**Impactor_06**	**Steel**	9.07	1.198	4.65	98.18	368.95	39.97	983	0.93	85	4600	40	61	938
**Impactor_09**	**Steel**	9.07	1.204	4.67	98.95	386.53	41.06	1061	0.94	118	4648	38	58	947
**Impactor_26**	**Steel**	12.78	0.816	3.94	99.04	318.11	45.17	870	0.98	67	4914	27	62	941
**Impactor_08**	**Steel**	9.07	1.205	4.68	99.24	391.34	40.97	1088	0.94	84	4645	39	58	951
**Impactor_10**	**Steel**	9.07	1.207	4.68	99.24	387.97	38.59	1068	0.94	150	4619	38	57	943
**Impactor_07**	**Steel**	9.07	1.203	4.68	99.28	371.65	39.25	999	0.93	52	4574	39	59	942
**Impactor_21**	**Steel**	12.78	0.851	4.04	104.30	353.42	52.45	1025	1.02	53	5163	29	64	1003
**Impactor_25**	**Steel**	12.78	0.98	4.31	118.87	385.54	53.53	1228	1.05	70	5336	31	65	1049
**Impactor_20**	**Steel**	12.78	1.064	4.46	127.11	407.07	52.36	1394	1.11	64	5700	34	67	1152
**Impactor_29**	**Steel**	12.78	1.174	4.68	139.78	480.17	58.45	1778	1.12	83	5592	34	69	1152
**Impactor_27**	**Steel**	12.78	1.315	4.94	155.62	532.12	81.42	2298	1.20	73	6037	37	76	1254
**Impactor_30**	**Steel**	12.78	1.514	5.28	178.14	585.16	71.86	2843	1.26	80	6207	39	82	1312

**Table 3:** Rigid impactor tests results – estimated severity of injury or probability of fatality.

internal ID	Impactor material	Impactor mass	Impact KE	Pof Head (KE-Feinstein)	PoF Head (KE-Janser)	Skull fracture AIS2 (Peak head acceleration Mertz)	Skull fracture AIS2 (HIC15 Mertz)	Skull fracture AIS2 (HIC36 NHTSA)	Head AIS 3 (HIC15 Prasad)	Head AIS 3 (HIC15 NHTSA)	Brain Injury AIS4 (HIC15 Prasad)	Brain Injury AIS4 (HIC15 Mertz)	AIS 3 injury (Nij)
Unit	–	[kg]	[J]	%	%	%	%	%	%	%	%	%	%
Limit value	–	–	80	100	100	100	100	100	100	100	100	100	100
**Impactor_12**	**Steel**	9.07	5.32	0.00	0.00	0.00	0.11	0.00	0.00	0.00	0.00	0.04	6.44
**Impactor_11**	**Steel**	9.07	10.29	0.00	0.00	0.00	0.12	0.00	0.00	0.00	0.00	0.05	7.56
**Impactor_13**	**Steel**	9.07	20.38	0.00	0.00	0.01	0.14	0.01	0.21	0.00	0.04	0.06	9.29
**Impactor_14**	**Steel**	9.07	30.28	0.06	0.00	0.10	0.21	0.29	1.46	0.01	0.32	0.10	10.77
**Impactor_15**	**Steel**	9.07	40.71	1.54	0.11	1.35	0.33	1.69	3.70	0.10	0.80	0.17	11.99
**Impactor_01**	**Steel**	9.07	47.84	5.66	0.58	1.44	0.39	2.53	4.70	0.19	1.01	0.21	13.48
**Impactor_02**	**Steel**	9.07	49.39	7.07	0.79	4.58	0.52	4.36	6.69	0.44	1.45	0.29	13.69
**Impactor_22**	**Steel**	12.78	49.56	7.24	0.81	2.03	0.42	2.96	5.18	0.24	1.12	0.23	14.40
**Impactor_04**	**Steel**	9.07	49.66	7.34	0.83	5.69	0.53	4.57	6.92	0.48	1.50	0.30	13.54
**Impactor_03**	**Steel**	9.07	49.90	7.58	0.86	4.85	0.50	4.19	6.52	0.42	1.41	0.28	13.41
**Impactor_05**	**Steel**	9.07	50.08	7.77	0.89	6.43	0.53	4.58	6.94	0.48	1.50	0.30	13.49
**Impactor_23**	**Steel**	12.78	50.10	7.79	0.90	2.53	0.47	3.67	5.96	0.34	1.29	0.26	14.65
**Impactor_16**	**Steel**	9.07	60.38	22.57	4.07	30.94	1.16	12.03	15.86	2.26	3.57	0.77	14.84
**Impactor_24**	**Steel**	12.78	69.59	40.25	10.23	29.54	1.60	15.85	21.54	3.55	5.03	1.13	17.44
**Impactor_17**	**Steel**	9.07	69.69	40.45	10.31	62.15	2.36	20.85	30.39	5.60	7.60	1.80	16.00
**Impactor_18**	**Steel**	9.07	79.46	58.98	20.48	88.06	5.03	31.23	52.99	11.11	16.80	4.36	17.45
**Impactor_19**	**Steel**	9.07	88.60	73.08	32.25	97.46	10.52	41.87	76.34	18.53	35.31	10.24	18.66
**Impactor_28**	**Steel**	12.78	97.19	82.78	43.99	97.90	15.03	47.13	85.22	22.88	48.59	15.36	21.49
**Impactor_06**	**Steel**	9.07	98.18	83.69	45.33	98.71	14.48	46.58	84.40	22.40	47.09	14.73	19.95
**Impactor_09**	**Steel**	9.07	98.95	84.36	46.36	99.53	18.43	50.18	89.22	25.61	57.08	19.30	20.26
**Impactor_26**	**Steel**	12.78	99.04	84.45	46.49	87.88	9.84	40.90	74.45	17.78	33.15	9.49	21.33
**Impactor_08**	**Steel**	9.07	99.24	84.62	46.76	99.65	19.94	51.35	90.55	26.71	60.43	21.06	20.26
**Impactor_10**	**Steel**	9.07	99.24	84.62	46.76	99.57	18.82	50.49	89.59	25.90	57.97	19.76	20.08
**Impactor_07**	**Steel**	9.07	99.28	84.65	46.81	98.88	15.24	47.34	85.52	23.06	49.15	15.60	19.93
**Impactor_21**	**Steel**	12.78	104.30	88.44	53.38	97.16	16.52	48.54	87.18	24.12	52.47	17.08	22.89
**Impactor_25**	**Steel**	12.78	118.87	95.19	69.92	99.50	28.84	57.00	95.32	32.32	75.70	31.57	24.03
**Impactor_20**	**Steel**	12.78	127.11	97.15	77.23	99.87	41.38	62.79	98.04	38.70	87.89	46.27	26.27
**Impactor_29**	**Steel**	12.78	139.78	98.75	85.64	100.00	71.58	73.04	99.75	51.68	98.08	78.84	26.50
**Impactor_27**	**Steel**	12.78	155.62	99.57	92.27	100.00	94.91	82.04	99.98	65.13	99.86	97.78	29.77
**Impactor_30**	**Steel**	12.78	178.14	99.91	96.96	100.00	99.70	87.87	100.00	75.06	99.99	99.95	32.05

The data show an interesting connection between KE and estimated HIC15, where after crossing the 80 J threshold (proposed limit within the EU legislation) [12], the 700 limit threshold (generally used limit defined either by the FMVSS [22] or Regulation No. 94. [23]) is also reached. However, the 3 ms head acceleration and upper neck compression results are not in agreement with the predicted severity from other performance criteria [22,23]. Only one test exceeded the safety threshold limit of 80 g or 6160 N.

Table 3 presents the estimated chance for severe injury or predicted PoF. The results show a gradual increase in the predicted injury severity with increasing KE. Overall, the prediction KE HVMs show a high rate of agreement with the automotive criterions and corresponding predicted injury severity. The gradual increase of the predicted injury severity or PoF proves the ability of the use of the ATD to assess the gradual increase of the impacting KE.

### Test validation

Validation of the test results was performed in three ways. The first was assessing the initial assumption that the rigid impactor is able to translate most of its impact KE to the ATD. Thus, there should be an obvious increase in the correspondence between the KE HVM (Feinstein/RCC, Janser) and automotive HVMs prediction. Table 4 shows the correlation (R^2^ determined through MS Excel data analysis correlation tool) for impactor tests. It is shows that the desired outcome was achieved. Interestingly, the level of correlation is lower (R^2^ of 0.68 and 0.69 compared to 0.88 and 0.89) for the initial Feinstein model used by RCC 321. The Janser model [11], not as commonly used, shows higher agreement with the automotive models’ predictions. However, it is important to note that while the KE HVM predicts the probability of fatality, the automotive predictions are only for the level of AIS injury occurrence (AIS 2–4). Thus, the results should be taken with these limitations in mind. On the other hand, the adjusted prediction models by Mertz show a lower level of correlation [24].

**Table 4:** Correlation between the HVMs for rigid impactor tests.

Correlation for rigid impactor tests (30 tests)	PoF RCC 321 Head (Feinstein)	PoF Janser Head	Skull fracture AIS2 (Peak head acceleration Mertz)	Skull fracture AIS2 (HIC15 Mertz)	Skull fracture AIS2 (HIC36 NHTSA)	Head AIS 3 (HIC15 Prasad)	Head AIS 3 (HIC15 NHTSA)	Brain Injury AIS4 (HIC15 Prasad)	Brain Injury AIS4 (HIC15 Mertz)	AIS 3 injury (Nij)
**PoF RCC 321 Head (Feinstein)**	1.00									
**PoF Janser Head**	0.93	1.00								
**Skull fracture AIS2 (Peak head acceleration Mertz)**	0.98	0.87	1.00							
**Skull fracture AIS2 (HIC15 Mertz)**	0.68	0.88	0.60	1.00						
**Skull fracture AIS2 (HIC36 NHTSA)**	0.96	0.98	0.93	0.85	1.00					
**Head AIS 3 (HIC15 Prasad)**	0.99	0.94	0.98	0.69	0.97	1.00				
**Head AIS 3 (HIC15 NHTSA)**	0.85	0.97	0.79	0.96	0.96	0.86	1.00			
**Brain Injury AIS4 (HIC15 Prasad)**	0.92	0.99	0.86	0.88	0.98	0.93	0.96	1.00		
**Brain Injury AIS4 (HIC15 Mertz)**	0.69	0.89	0.60	1.00	0.85	0.70	0.96	0.89	1.00	
**AIS 3 injury (Nij)**	0.91	0.95	0.85	0.84	0.95	0.90	0.93	0.93	0.85	**1.00**

Notable is the 80 J energy impact test which led to a value of HIC15 of 699. This is interesting in several aspects. While the setting of the 80 J was as a 20–30% chance of fatality and was determined based on the KE HVMs, the 700 HIC15 threshold value is connected with a severity of 17%, resp. 5%, for AIS 4 + brain injury. Even though the AIS scale can be related to the probability of fatality [25] as 0.1–0.4% for AIS 2; 0.8–2.1% for AIS 3 and 7.9–10.6% for AIS 4, the resulting PoF is still significantly lower. As the most probable answer, the difference in base datasets and the design of these prediction models can be seen. For KE HVM, small, lightweight, and very fast objects were considered, while for the automotive industry, the human is impacting a solid structure of the inside of a vehicle with relatively lower speeds. Thus, a direct agreement may never be found. Nevertheless, the HIC15 value limit near the 80 J energy impact seems to confirm the suitability of the 80 J limit set as the threshold value for the energy transmitted to the human body. If this impact energy is taken as a measure that divides potentially safe and hazardous impacts, the Prasad-based AIS 3 predictive function (with a 53% chance of serious injury occurring) seems more suitable.

The second validation was based on the estimation of standard deviations of measured values for the same KE. The standard deviation was determined not only for each group of tests with the same weight of the impactor but also for all the tests combined (Table 5 and Table 6). The standard deviations were also related to the threshold limits and to the mean value of all tests. It can be said that the variation is higher for lower measured values, which can be expected. While the highest variation was observed for the peak head acceleration, all of the tests show the measured values in similar ranges and none of them deviate. Furthermore, based on the data, the differences in the observed response of the ATD do not differ due to the increased weight of the impactor.

**Table 5:** Standard deviations for the 50 J rigid impactor tests.

Internal ID	UAS model	UAS mass	Impact velocity	Impact KE	Maximum Resultant Head Acceleration	Head Accel. (3ms)	Head injury criterion (HIC15)	Neck injury criterion (Nij)	Upper neck tension (Fz)	Upper neck compression (-Fz)	Upper neck flexion (-My)	Upper neck extension (My)	Upper neck shear
Unit	–	kg	m/s	J	(g)	(g)	–	–	N	N	Nm	Nm	N
Limit value	–	–	–	80	200	80	700	1	6806	6160	310	135	3100
**Impactor_01**	**Impactor 1**	9.07	3.25	47.84	157.08	21.17	202	0.69	32	3447	29	49	635
**Impactor_02**	**Impactor 1**	9.07	3.30	49.39	181.01	25.74	248	0.70	48	3511	31	48	652
**Impactor_04**	**Impactor 1**	9.07	3.31	49.66	186.09	27.30	253	0.70	24	3470	31	49	646
**Impactor_03**	**Impactor 1**	9.07	3.32	49.90	182.35	26.26	245	0.69	108	3454	30	47	643
**Impactor_05**	**Impactor 1**	9.07	3.32	50.08	189.05	26.88	254	0.70	25	3456	31	48	654
**Impactor_22**	**Impactor 2**	12.78	2.79	49.56	163.68	22.90	214	0.73	45	3671	12	48	656
**Impactor_23**	**Impactor 2**	12.78	2.80	50.10	168.18	24.54	232	0.74	30	3749	13	47	670
**Mean**			3.15	49.50	174.97	24.88	234.68	0.71	38.97	3535.05	23.65	48.21	650.59
**Standard deviation for Impactor 1**			**0.03**	**0.89**	**12.71**	**2.48**	**21.71**	**0.00**	**35.21**	**25.53**	**0.62**	**0.87**	**7.82**
**Standard deviation related to limit**			**–**	**–**	**6.36%**	**3.10%**	**3.10%**	**0.46%**	**0.52%**	**0.41%**	**0.20%**	**0.64%**	**0.25%**
**Standard deviation for Impactor 2**			**0.01**	**0.38**	**3.18**	**1.17**	**12.94**	**0.01**	**10.78**	**55.45**	**0.52**	**0.45**	**9.95**
**Standard deviation related to limit**			**–**	**0.77%**	**1.76%**	**4.53%**	**5.21%**	**1.01%**	**22.55%**	**1.58%**	**1.69%**	**0.93%**	**1.53%**
**Standard deviation (all tests)**			**0.25**	**0.78**	**12.28**	**2.25**	**20.31**	**0.02**	**29.46**	**122.31**	**8.77**	**0.80**	**11.16**
**Standard deviation related to limit**			**–**	**–**	**6.14%**	**2.81%**	**2.90%**	**2.12%**	**0.43%**	**1.99%**	**2.83%**	**0.60%**	**0.36%**
**Standard deviation related to mean value**			**–**	**1.57%**	**7.02%**	**9.03%**	**8.65%**	**2.99%**	**75.60%**	**3.46%**	**37.07%**	**1.67%**	**1.71%**

**Table 6:** Standard deviations for the 100 J rigid impactor tests.

internal ID	UAS model	UAS mass	Impact velocity	Impact KE	Maximum Resultant Head Acceleration	Head Accel. (3ms)	Head injury criterion (HIC15)	Neck injury criterion (Nij)	Upper neck tension (Fz)	Upper neck compression (-Fz)	Upper neck flexion (-My)	Upper neck extension (My)	Upper neck shear
Unit	–	kg	m/s	J	(g)	(g)	–	–	N	N	Nm	Nm	N
Limit value	–	–	–	80	200	80	700	1	6806	6160	310	135	3100
**Impactor_06**	**Impactor 1**	9.07	4.65	98.18	368.95	39.97	983	0.93	85	4600	40	61	938
**Impactor_09**	**Impactor 1**	9.07	4.67	98.95	386.53	41.06	1061	0.94	118	4648	38	58	947
**Impactor_08**	**Impactor 1**	9.07	4.68	99.24	391.34	40.97	1088	0.94	84	4645	39	58	951
**Impactor_10**	**Impactor 1**	9.07	4.68	99.24	387.97	38.59	1068	0.94	150	4619	38	57	943
**Impactor_07**	**Impactor 1**	9.07	4.68	99.28	371.65	39.25	999	0.93	52	4574	39	59	942
**Impactor_26**	**Impactor 2**	12.78	3.94	99.04	318.11	45.17	870	0.98	67	4914	27	62	941
**Impactor_28**	**Impactor 2**	12.78	3.90	97.19	359.62	48.41	995	0.98	64	4986	28	60	975
**Mean**			4.44	98.73	368.38	41.79	1006.92	0.95	83.43	4709.68	35.15	59.28	948.13
**Standard deviation for Impactor 1**			**0.01**	**0.46**	**10.23**	**1.07**	**46.00**	**0.01**	**37.46**	**31.07**	**0.71**	**1.55**	**5.01**
**Standard deviation related to limit**			**–**	**–**	**5.11%**	**1.34%**	**6.57%**	**0.51%**	**0.55%**	**0.50%**	**0.23%**	**1.15%**	**0.16%**
**Standard deviation for Impactor 2**			**0.03**	**1.31**	**29.35**	**2.30**	**88.16**	**0.00**	**2.05**	**50.84**	**0.63**	**0.98**	**24.00**
**Standard deviation related to limit**			**–**	**1.32%**	**7.59%**	**5.59%**	**8.31%**	**0.36%**	**1.74%**	**1.09%**	**1.66%**	**1.69%**	**2.53%**
**Standard deviation (all tests)**			**0.37**	**0.78**	**25.33**	**3.57**	**73.90**	**0.02**	**34.32**	**165.56**	**5.52**	**1.81**	**12.63**
**Standard deviation related to limit**			**–**	**–**	**12.67%**	**4.46%**	**10.56%**	**2.03%**	**0.50%**	**2.69%**	**1.78%**	**1.34%**	**0.41%**
**Standard deviation related to mean value**			**–**	**0.79%**	**6.88%**	**8.53%**	**7.34%**	**2.14%**	**41.14%**	**3.52%**	**15.71%**	**3.05%**	**1.33%**

As a third validation method, a comparison with data from the database was selected. Steel, wooden, and foam blocks together with DJI Phantom 3 battery impact tests were available for comparison (from the Assure A4 and A14 reports [5,6]). The comparison showed that the measured values significantly differed from those acquired in this study, in some cases by an order of magnitude. There were no additional tests that could clarify this difference. However, the tests performed on wooden blocks impacting PMHS [26] (while performed for horizontal impacts) showed values closer to the results of rigid impacts performed in the Assure ATD testing. The actual values of HIC15 were not stated in the report. The values ranged from approximately 600–3400 HIC15.

Furthermore, additional impact tests were performed with a changed orientation of the CG of ATD head in relation to the body. The main aim was to determine potential differences in the resulting values. The tests were performed with identical settings and apparatus, but the position of the CG of the ATD was changed to a more prone position. In total, eleven tests were performed with an impactor weight of 12.78 kg – three for 50 J, three for 100 J, three for 150 J, and two for 180 J KE. The tests results are summarised in the following Table 7.

**Table 7:** Supplementary rigid impactor tests results (changed position of the CG of the head) – measured values.

internal ID	UAS model	UAS mass	Heigth	Impact velocity	Impact KE	Maximum Resultant Head Acceleration	Head Accel. (3ms)	Head injury criterion (HIC15)	Neck injury criterion (Nij)	Upper neck tension (Fz)	Upper neck compression (-Fz)	Upper neck flexion (-My)	Upper neck extension (My)	Upper neck shear
Unit	–	kg	m	m/s	J	(g)	(g)	–	–	N	N	Nm	Nm	N
Limit value	–	–	–	–	80	200	80	700	1	6806	6160	310	135	3100
Impactor_36	Impactor 2	12.780	0.416	2.80	49.99	240.86	8.73	397	0.77	235	4300	23	10	761
Impactor_32	Impactor 2	12.780	0.418	2.80	50.10	233.70	8.16	393	0.75	224	4258	20	14	747
Impactor_35	Impactor 2	12.780	0.419	2.81	50.28	245.84	7.39	410	0.75	310	4311	17	6	778
Impactor_34	Impactor 2	12.780	0.841	3.93	98.69	438.37	11.64	1418	1.01	310	5672	27	24	1063
Impactor_33	Impactor 2	12.780	0.842	3.96	99.95	414.36	13.90	1325	1.01	308	5664	29	24	1061
Impactor_31	Impactor 2	12.780	0.84	4.00	102.24	389.60	11.75	1152	1.01	183	5632	30	22	1049
Impactor_38	Impactor 2	12.780	1.249	4.78	146.25	604.16	15.18	2977	1.21	246	6789	37	14	1340
Impactor_39	Impactor 2	12.780	1.252	4.81	147.84	625.57	25.99	3144	1.22	206	6890	35	10	1357
Impactor_37	Impactor 2	12.780	1.254	4.87	151.55	538.15	17.48	2402	1.19	277	6578	41	20	1267
Impactor_41	Impactor 2	12.780	1.508	5.28	177.94	725.00	17.08	4391	1.34	243	7755	33	21	1505
Impactor_40	Impactor 2	12.780	1.509	5.32	180.65	726.26	21.50	4444	1.36	270	7747	37	14	1494

**Fig 2:**
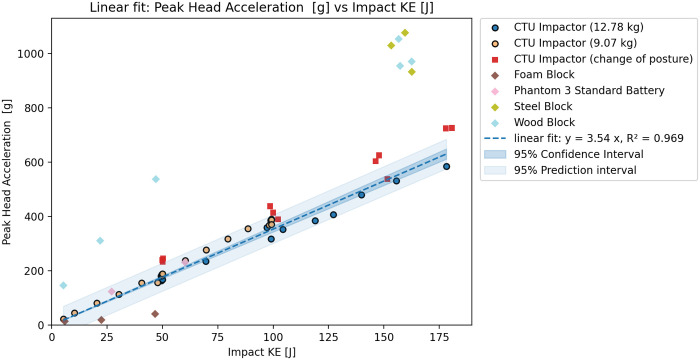
Rigid impactor tests results – Relation between Peak head acceleration and KE.

**Fig 3:**
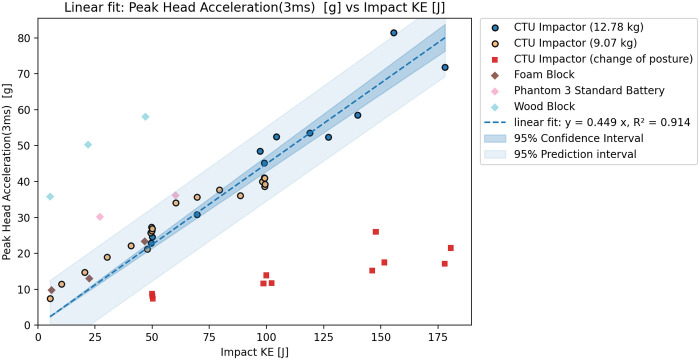
Rigid impactor tests results – Relation between Peak head acceleration (3 ms) and KE.

**Fig 4:**
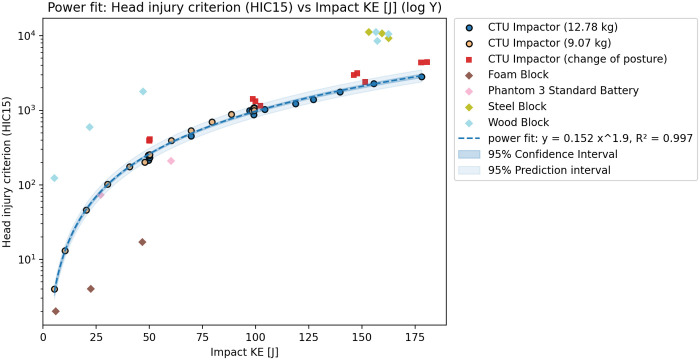
Rigid impactor tests results – Relation between HIC_15_ and KE.

**Fig 5:**
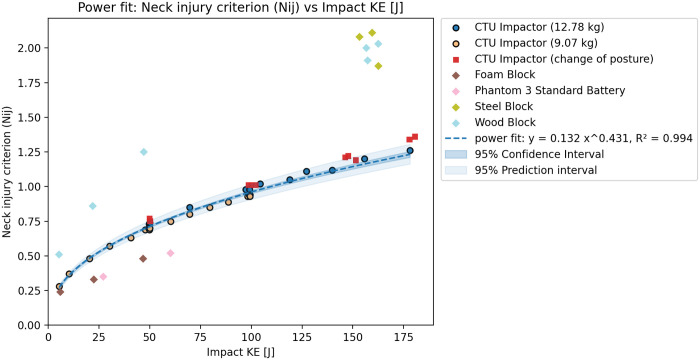
Rigid impactor tests results – Relation between N_ij_ and KE.

**Fig 6:**
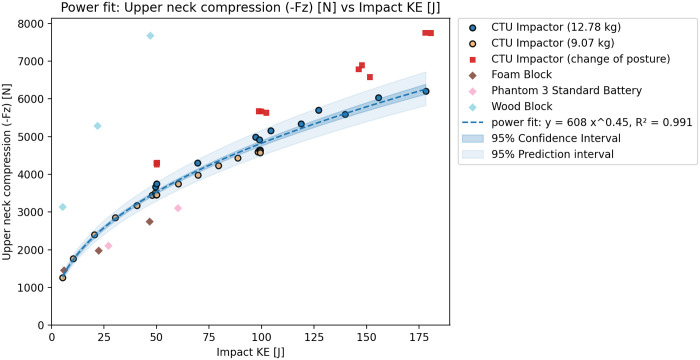
Rigid impactor tests results – Relation between Upper Neck Compression -F_z_ and KE.

The test results agreed with the initial tests when compared. However, a significant difference could be seen in the 3 ms head acceleration (Fig 2), which was lower for all tests with this configuration (Fig 3). Based on the results, the integration of the acceleration over time - HIC15 (Fig 4) seems to show less variation due to changes in the CG of the head. The additional tests however did not lead to significant differences in the observer values of peak head acceleration or the HIC15. Subsequently, it is also possible to see the relations between Nij and KE (Fig 5), and also between Upper Neck Compression (F_Z_) and KE (Fig 6).

### Test results

Based on the tests and validation results, the initial dataset of rigid impactor tests with the identical position of the ATD was used to show the relation between impact KE and peak head acceleration, peak head acceleration over 3ms, HIC15, Nij and Upper neck compression. The relation between peak head acceleration and rigid impactor appears to be linear, which agrees with the assumptions of the Assure Group. The HIC15, Nij and upper neck compression seem to be best described by a power fit. The graphs show that, while the correspondence is rather high for HIC15 and Nij HVMs, the peak head acceleration, and even more so the peak acceleration over 3ms, show higher variation despite clearly following an easily definable trend. The variation is most likely caused by even slight differences in testing scenarios, such as posture of the ATD or impacting mass. This can be shown through R^2^ score – for 3ms peak head acceleration is overall fit for all tests 0.914, 0.796 for lower mass impactor, 0.910 for higher mass impactor and only 0.094 with change of posture. On the other hand, for the HIC15 is overall R^2^ score 0.997, with value of 0.977 for the lower mass impactor, 0.991 for the higher mass impactor and 0.701 with changed posture. Despite this, the aggregated results show resulting R^2^s above 0.91 for all cases, thus, suggest an overall good fit of the data.

The 3 ms peak head acceleration, and upper neck compression results are not in agreement with the predicted severity from other performance criteria. While the Peak head acceleration, HIC15 and Nij are clearly showing progressively increasing severity through the whole range between 5-180J, the 3ms and neck compression exceeded their limit only at very high KE impacts and are overall showing lower values for this test configuration (vertical impact to the top of the head). As a result, for the subsequent phase of the safety assessment, only the peak head acceleration, HIC15 and Nij relations to KE were used for the maximum transmitted energy estimation.

If all of the aforementioned test results are combined with data measured in a drone crash test, the data allows for a comprehensive assessment of the impact consequences. The results of the impactor testing can be used to describe the amount of transferred kinetic energy, which can be subsequently applied to resolve discrepancies between kinetic energy models and automotive criteria. Therefore, along with the information about the terminal velocity and corresponding impact tests, it enables an alternative way to describe the consequences of a potential collision with a human.

As an example of the generalization, the tests for DJI Phantom Series drones can be used to demonstrate the advantages of the complementary dataset. Considering the limitations of the scope of this study – vertical impacts to the head. The most probable worst-case scenario resulting into the vertical impact to the head should be based on the four-motor failure in hover.

For this particular drone type (all DJI Phantom series drones), a total of 24 tests are available for the vertical impact of the bottom of the drone to the top of the ATD’s head (KE ranging from 35 to 280 J). The DFET tests were performed for higher impact KE to supplement the Assure dataset [9]. If the results are compared based on the resulting values of the observer vulnerability criteria (peak head acceleration, HIC15, Nij) with the corresponding values from the impactor testing, the maximum transferred kinetic energy during the impact can be indirectly determined.

The comparison with impactor tests shows that, although the UAS is potentially able to reach a KE of up to 280 J at a limit speed of 20 m/s, the transmitted energy is only about 20%. If the derived relations are used (Table 8, Table 9), the estimated maximum transmitted energy from all of the tests is 52,18 J.

**Table 8:** DJI Phantom series tests (vertical impact, bottom to head) – Impact parameters of comparable tests from the database, maximum values of criterions and the estimated transmitted energy.

Internal ID	UAS model	UAS mass	Impact velocity	Impact KE	Maximum Resultant Head Acceleration	Acceleration 3ms	HIC15	Nij
Unit	–	kg	m/s	J	(g)	(g)	–	–
Limit value	–	–	–	80	200	80	700	1
**CTU_21_38**	**DJI Phantom 1**	1.265	15.10	144.16	103.98	20.79	45.81	0.58
**CTU_21_39**	**DJI Phantom 3 Standard**	1.224	15.07	138.98	58.90	26.94	43.26	0.55
**CTU_21_40**	**DJI Phantom 3 Standard**	1.216	17.96	196.08	121.62	30.90	107.70	0.70
**CTU_21_41**	**DJI Phantom 2V+**	1.265	18.21	209.70	119.54	21.84	119.50	0.06
**CTU_21_42**	**DJI Phantom 2V+**	1.265	19.92	250.90	130.05	21.00	116.45	0.68
**CTU_21_43**	**DJI Phantom 4 Pro**	1.380	14.83	151.82	55.48	18.92	23.88	0.39
**CTU_21_44**	**DJI Phantom 4 Pro**	1.386	17.65	215.95	70.70	24.18	56.79	0.57
**CTU_21_45**	**DJI Phantom 4 Pro**	1.382	20.10	279.09	89.24	37.46	106.10	0.71
**CTU_21_46**	**DJI Phantom 4 Pro**	1.382	18.00	223.88	73.31	48.86	136.58	0.73
**UA17A-01**	**DJI Phantom 3 Standard**	1.220	9.90	59.83	54.31	0.00	12.02	0.42
**UA17A-02**	**DJI Phantom 3 Standard**	1.225	9.85	59.39	56.68	0.00	14.99	0.45
**UA17A-03**	**DJI Phantom 3 Standard**	1.225	9.91	60.09	49.18	0.00	15.64	0.47
**UA17A-04**	**DJI Phantom 3 Standard**	1.220	11.97	87.36	45.95	0.00	13.38	0.50
**UA17A-05**	**DJI Phantom 3 Standard**	1.225	11.96	87.64	47.78	0.00	19.29	0.48
**UA17A-06**	**DJI Phantom 3 Standard**	1.220	11.89	86.21	48.35	0.00	23.45	0.56
**UA17A-07**	**DJI Phantom 3 Standard**	1.218	11.81	84.90	66.36	0.00	23.02	0.46
**UA17A-08**	**DJI Phantom 3 Standard**	1.220	13.13	105.19	78.70	0.00	46.62	0.63
**UA17A-09**	**DJI Phantom 3 Standard**	1.220	13.17	105.82	54.01	0.00	34.13	0.65
**UA17A-10**	**DJI Phantom 3 Standard**	1.220	13.40	109.53	78.62	0.00	42.79	0.57
**UA17A-11**	**DJI Phantom 3 Standard**	1.222	15.11	139.58	82.38	0.00	59.54	0.65
**UA17A-12**	**DJI Phantom 3 Standard**	1.220	14.99	137.03	71.50	0.00	48.04	0.62
**UA17A-13**	**DJI Phantom 3 Standard**	1.211	14.98	135.85	119.13	0.00	42.24	0.63
**UA19A-21**	**DJI Phantom 3 Standard**	1.218	7.67	35.84	21.81	14.19	4.83	0.32
**UA19A-22**	**DJI Phantom 3 Standard**	1.220	11.07	74.73	27.90	16.89	7.92	0.37
Maximum value				279.09	130.05	48.86	130.05	0.73
**Estimated transmitted KE based on max value (KE [J])**					**36.75**		**35.69**	**52.18**

**Table 9:** DJI Phantom series tests (vertical impact, bottom to head) – Maximum values of criterions and the percentage of estimated transmitted energy.

Internal ID	Maximum Resultant Head Acceleration	HIC15	Nij	Estimated transmitted energy based on peak head acceleration	% of Estimated transmitted energy based on peak head acceleration	Estimated transmitted energy based on HIC	% of estimated transmitted energy based on HIC	Estimated transmitted energy based on Nij	% of estimated transmitted energy based on Nij
Unit	[g]	–	–	–	%	–	%	–	%
Limit value	200	700	1	80	100	700	100	80	100
**UA19A-21**	21.81	4.83	0.32	**6.16**	**17.23**	**6.18**	**17.26**	**7.81**	**21.83**
**UA17A-02**	56.68	14.99	0.45	**16.02**	**27.22**	**11.19**	**19.02**	**17.20**	**29.23**
**UA17A-01**	54.31	12.02	0.42	**15.35**	**25.84**	**9.97**	**16.78**	**14.66**	**24.69**
**UA17A-03**	49.18	15.64	0.47	**13.90**	**23.34**	**11.44**	**19.22**	**19.02**	**31.95**
**UA19A-22**	27.900	7.92	0.37	**7.88**	**10.59**	**8.01**	**10.76**	**10.93**	**14.68**
**UA17A-07**	66.36	23.02	0.46	**18.75**	**22.13**	**14.02**	**16.54**	**18.10**	**21.36**
**UA17A-06**	48.35	23.45	0.56	**13.66**	**15.94**	**14.16**	**16.51**	**28.54**	**33.29**
**UA17A-05**	47.78	19.29	0.48	**13.50**	**15.55**	**12.78**	**14.71**	**19.97**	**23.00**
**UA17A-04**	45.95	13.38	0.50	**12.98**	**14.95**	**10.54**	**12.14**	**21.95**	**25.27**
**UA17A-08**	78.70	46.62	0.63	**22.24**	**21.22**	**20.30**	**19.37**	**37.49**	**35.77**
**UA17A-09**	54.01	34.13	0.65	**15.26**	**14.53**	**17.24**	**16.41**	**40.30**	**38.37**
**UA17A-10**	78.62	42.79	0.57	**22.21**	**20.43**	**19.41**	**17.85**	**29.73**	**27.35**
**UA17A-13**	119.13	42.24	0.63	**33.66**	**24.78**	**19.28**	**14.19**	**37.49**	**27.59**
**UA17A-12**	71.50	48.04	0.62	**20.20**	**14.83**	**20.63**	**15.14**	**36.12**	**26.51**
**UA17A-11**	82.38	59.54	0.65	**23.28**	**16.83**	**23.08**	**16.69**	**40.30**	**29.14**
**CTU_21_39**	58.90	43.26	0.55	**16.64**	**11.97**	**19.52**	**14.05**	**26.87**	**19.33**
**CTU_21_38**	103.98	45.81	0.58	**29.38**	**20.38**	**20.12**	**13.95**	**31.33**	**21.73**
**CTU_21_43**	55.48	23.88	0.39	**15.68**	**10.33**	**14.29**	**9.41**	**12.05**	**7.94**
**CTU_21_40**	121.62	107.70	0.70	**34.37**	**17.53**	**31.51**	**16.07**	**47.56**	**24.26**
**CTU_21_41**	119.54	119.50	0.06	**33.78**	**16.11**	**33.28**	**15.87**	**0.19**	**0.09**
**CTU_21_44**	70.70	56.79	0.57	**19.98**	**9.25**	**22.52**	**10.43**	**30.31**	**14.04**
**CTU_21_46**	73.31	136.58	0.73	**20.71**	**9.25**	**35.69**	**15.94**	**52.18**	**23.31**
**CTU_21_42**	130.05	116.45	0.68	**36.75**	**14.65**	**32.83**	**13.08**	**44.40**	**17.70**
**CTU_21_45**	89.24	106.10	0.71	**25.21**	**9.03**	**31.26**	**11.20**	**49.14**	**17.61**
**Estimated transmitted KE based on max value**	**48.86**	**136.58**	**0.73**		**16.83%**		**15.11%**		**23.17%**

While this may seem counterintuitive, the drone is impacting the top of the head with a metal camera, and the results are clearly showing that the elastic and plastic deformations of the UAS frame are able to absorb a significant amount of kinetic energy. It is important to note that the deformations of the gimbal and camera may lead to sharp metallic fragments and cause other types of injuries, such as penetration or laceration. These are, however, outside the scope and limitations of this study.

**Fig 7:**
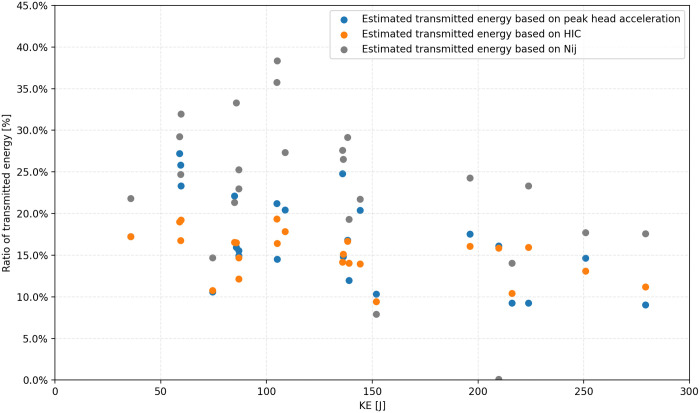
Estimated ratio of the maximum transmitted energy to the head for impact kinetic energy for vertical impacts of the bottom of the DJI Phantom to the top of the head, based on selected biomechanical criteria.

An interesting aspect of the analyzed data is the changes in the estimated maximum transmitted energy for various impact speeds of the UAS. It can be seen that the amount (percentage) of the transmitted energy is slowly decreasing with increasing impact KE (Fig 7). This can be attributed to the fragility of the DJI Phantom drone design and materials. The plastic frame is undergoing heavy plastic deformations and seems unable to transmit the increasing energy to the head. This trend is also confirmed by the observed biomechanical criteria (Peak Head Acceleration, HIC15, and Nij), where it is seen that with increasing kinetic energy, its values gradually approach a certain value specific to the drone.

Similarly, the estimation of the maximum transmitted energy can be done for the DJI Mavic series drones. While the tests consist of impacts of DJI Mavic Pro and Mavic 2 Pro, which have different mass, the design characteristics are very similar (Table 10).

**Table 10:** DJI Mavic series tests (vertical) – Aggregated impact parameters of the performed test, comparable tests from the database, maximum values of criterions and the estimated maximum transmitted energy.

Internal ID	UAS model	UAS mass	Vehicle Orientation to ATD	Impact velocity	Impact KE	Maximum Resultant Head Acceleration	Acceleration 3ms	HIC15	Nij	Estimated transmitted KE			Ratio of Transmitted Energy		
										a	HIC15	Nij	a	HIC15	Nij
Unit	–	kg		m/s	J	(g)	(g)	–	–	–	–	–	%	%	%
Limit value					80	200	80	700	1	80	80	80	100	100	100
CTU_21_35	DJI Mavic Pro	0.744	Bottom Into Head	18.14	122.40	168.50	30.62	186	0.61	47.61	41.97	35.18	38.90	34.29	28.75
CTU_21_31	DJI Mavic Pro 2	0.917	Bottom Into Head	17.99	148.32	235.40	31.00	356	0.76	66.51	59.03	58.33	44.84	39.80	39.33
CTU_21_32	DJI Mavic Pro 2	0.918	Bottom Into Head	20.08	185.13	302.47	35.87	560	0.84	85.46	74.85	72.61	46.16	40.43	39.22
CTU_21_36	DJI Mavic Pro 2	0.920	Bottom Into Head	18.13	151.12	247.32	31.99	450	0.73	69.88	66.73	51.91	46.24	44.16	34.35
CTU_21_37	DJI Mavic Pro 2	0.913	Bottom Into Head	18.08	149.28	241.95	36.82	393	0.73	68.37	62.20	52.94	45.80	41.67	35.46
CTU_21_49	DJI Mavic Pro 2	0.920	Bottom Into Head	18.06	149.96	191.37	29.48	245	0.49	54.07	48.52	20.56	36.06	32.36	13.71
CTU_21_34	DJI Mavic Pro	0.750	Top Into Head	18.22	124.52	246.94	22.67	263	0.66	69.78	50.39	41.43	56.04	40.47	33.27
CTU_21_33	DJI Mavic Pro 2	0.914	Top Into Head	19.88	180.52	279.04	44.64	660	0.88	78.84	81.59	80.60	43.68	45.20	44.65
UA19A-76	DJI Mavic Pro	0.699	Top Into Head	18.19	115.68	241.54	51.10	298	0.59	68.25	53.77	32.20	59.00	46.48	27.84
UA19A-75	DJI Mavic Pro	0.711	Top Into Head	15.60	86.54	285.72	48.33	325	0.58	80.73	56.29	30.95	93.29	65.05	35.77

Other various types of lightweight UASs were tested in this study to estimate the effects of design and material characteristics on the ratio of transmitted energy. Table 11 summarizes the estimated transmitted energy based on biomechanical criteria for lightweight UASs (under 0.5 kg).

**Table 11:** Lightweight UAS tests (vertical) – Aggregated impact parameters of the performed test, comparable tests from the database, maximum values of criterions and estimated transmitted energy and its ratio to impact KE based on peak head acceleration, HIC15 and Nij criterions.

Internal ID	UAS model	UAS mass	Vehicle Orientation to ATD	Impact velocity	Impact KE	Maximum Resultant Head Acceleration	Acceleration 3ms	HIC15	Nij	Estimated transmitted KE			Ratio of Transmitted Energy		
										a	HIC 15	Nij	a	HIC15	Nij
Unit	–	kg	–	m/s	J	(g)	(g)	–	–	–	–	–	%	%	%
Limit value	–	–	–	–	80	200	80	700	1	80	80	80	100	100	100
**CTU_21_03**	**Tiny Whoop**	0.028	Side Into Head	15.97	3.57	3.31	0.68	0	0.02	0.93	0.30	0.01	26.19	8.29	0.29
**CTU_21_04**	**Tiny Whoop**	0.028	Bottom Into Head	13.50	2.55	5.62	0.46	0	0.00	**1.59**	0.38	0.00	62.20	14.91	0.01
**CTU_21_06**	**DJI Tello**	0.086	Bottom Into Head	16.33	11.47	58.69	6.94	7	0.13	16.58	7.34	0.95	**144.61**	64.01	8.26
**CTU_21_08**	**DJI Tello**	0.086	Bottom Into Head	17.81	13.65	84.19	3.33	14	0.16	23.79	10.62	1.54	**174.33**	77.86	11.31
**CTU_21_10**	**Eachine E58**	0.089	Bottom Into Head	11.67	6.06	18.92	3.51	1	0.06	5.35	1.91	0.13	88.28	31.48	2.20
**CTU_21_11**	**Eachine E58**	0.089	Bottom Into Head	15.77	11.06	36.75	2.09	2	0.07	10.38	3.75	0.26	93.86	33.90	2.37
**CTU_21_14**	**Syma X5C**	0.099	Bottom Into Head	12.63	7.89	11.01	2.57	0	0.07	3.11	1.45	0.24	39.41	18.43	3.10
**CTU_21_15**	**Syma X5C**	0.099	Bottom Into Head	18.25	16.48	20.60	4.62	1	0.11	5.82	3.09	0.64	35.31	18.76	3.89
**CTU_21_16**	**Syma X5C**	0.099	Bottom Into Head	18.35	16.67	17.95	4.51	1	0.10	5.07	3.16	0.55	30.43	18.96	3.32
**CTU_21_17**	**Syma X5C**	0.099	Bottom Into Head	18.48	16.90	23.69	5.67	1	0.11	6.69	2.94	0.64	39.61	17.38	3.80
**CTU_21_28**	**DW686**	0.120	Bottom Into Head	13.72	11.30	11.76	1.86	0	0.07	3.32	1.17	0.21	29.40	10.32	1.84
**CTU_21_21**	**RR Meffie Nano**	0.131	Bottom Into Head	17.12	19.20	68.20	6.29	11	0.12	19.27	9.65	0.79	**100.39**	50.26	4.13
**CTU_21_22**	**RR Meffie Nano**	0.131	Bottom Into Head	23.89	37.38	63.62	11.89	17	0.18	17.98	12.02	2.01	48.09	32.15	5.37
**CTU_21_26**	**FOYU FO-F708**	0.149	Bottom Into Head	14.02	14.65	20.51	3.79	1	0.07	5.80	2.56	0.26	39.57	17.50	1.80
**CTU_21_18**	**Eachine E520s**	0.238	Bottom Into Head	13.03	20.21	28.47	8.66	4	0.14	8.04	5.62	1.18	39.79	27.80	5.83
**CTU_21_19**	**Eachine E520s**	0.239	Bottom Into Head	19.81	46.89	55.97	12.77	12	0.21	15.81	9.90	2.85	33.73	21.11	6.08
**CTU_21_20**	**Eachine E520s**	0.238	Bottom Into Head	23.49	65.66	67.46	13.09	17	0.24	19.06	12.09	4.00	29.03	18.42	6.10
**CTU_21_23**	**RR Calypso**	0.298	Bottom Into Head	20.04	59.83	104.25	20.30	47	0.36	29.46	20.29	9.97	49.23	33.92	16.67
**CTU_21_27**	**RR Calypso**	0.298	Bottom Into Head	18.97	53.63	79.11	7.14	28	0.34	22.35	15.39	8.86	41.68	28.70	16.51
**CTU_21_29**	**DJI Spark**	0.305	Bottom Into Head	18.00	49.41	173.46	12.90	129	0.35	49.01	34.66	9.64	99.20	70.15	19.50
**UA19A-08**	**Vendor 1**	0.323	Arm Into Head	11.14	20.04	14.89	7.85	1	0.10	4.21	2.79	0.53	20.99	13.90	2.64
**UA19A-02**	**Vendor 1**	0.322	Bottom Into Head	10.88	19.04	23.63	8.87	2	0.11	6.68	3.48	0.66	35.06	18.28	3.46
**UA19A-06**	**Vendor 1**	0.321	Side Into Head	11.08	19.71	15.62	11.23	2	0.18	4.41	4.21	2.06	22.39	21.37	10.46
**UA19A-18**	**Vendor 1**	0.323	Top Into Head	10.94	19.32	67.87	16.79	13	0.21	19.18	10.24	2.95	99.24	53.00	15.24
**UA19A-20**	**Vendor 1**	0.324	Top Into Head	16.76	45.49	145.20	26.62	48	0.34	41.03	20.52	8.99	90.19	45.11	19.76
**CTU_21_30**	**DJI Air**	0.435	Bottom Into Head	18.32	72.99	271.41	23.48	265	0.51	76.69	50.53	23.07	**105.06**	69.23	31.61
**CTU_21_24**	**Rotorama Cinewhoop**	0.485	Bottom Into Head	15.66	59.46	108.42	25.10	62	0.44	30.63	23.65	16.29	51.52	39.77	27.40
**CTU_21_25**	**Rotorama Cinewhoop**	0.485	Bottom Into Head	20.13	98.22	170.05	17.43	169	0.52	48.05	39.96	23.60	48.92	40.68	24.03

All tests, apart from the test with Rotorama CineWhoop (CTU_21_25), had an impact KE under 80 J. Furthermore, only the test with DJI Air (test CTU_21_30) exceeded a threshold value for measured biomechanical criteria (271 g for peak head acceleration).

The results show significant differences in the estimated transmitted energy, based on the chosen biomechanical criteria. Some of these differences can be attributed to the induced loads by the impact (such as higher load to the head or neck during the crash and post-crash behaviour of the drone). Meanwhile, others can be attributed to the variance of the measured data for low impact KE (caused by only a minimal response from the ATD). Therefore, based on the measured values, observed ATD response and subsequent impact footage analysis, the most important loadings and corresponding estimated energy via rational analysis must be selected. However, the potential problem presents the estimation based on peak head acceleration, where the estimated energy is higher than the impact KE.

The results suggest that the proposed methodology is more suitable for higher KE impacts, where the response from the ATD is sufficient. As seen for the DJI Phantom and Mavic drones, for higher KE, the method is able to estimate the transmitted energy rather consistently. In the case of the DJI Phantom, this behavior can be attributed to its specific design characteristics, in particular its relatively low structural density, highly deformable plastic frame, and large frontal area, which promote significant structural deformation upon impact. These characteristics reduce the effective energy transfer compared to more compact and rigid UAS designs. Its accuracy and applicability are directly determined by the selected range of KE for comparative impactor tests (5 - 180J with 10 J steps). Nevertheless, this should not affect its usability due to the necessity to perform assessments and risk estimations mainly for UAS with impact KE above 80 J.

Despite these limitations, the HIC-based prediction of transmitted energy was utilised for the generalised assessment of lightweight UASs. It can be seen that similarly to DJI Mavic drones, DJI Tello, Spark and Air can transmit a higher ratio of energy due to their compact design (approx. 70%). On the other hand, less compact hobby drones with lower body density, such as Syma X5C, FOYUFO F708 or Eachine E520s, transmit less energy to the head (approx. 20%) thanks to the frangibility and deformability of their body structure. Interestingly, the camera placed at the bottom of the drone (Syma X5C) led to a lower transmission of energy than in the case of a drone with the camera at the front (Eachine E58) – 20 vs 30%.

## Discussion

The proposed approach demonstrates that rigid impactor tests can be effectively used to indirectly estimate the transmitted kinetic energy. This enables assessing safety risks based on available crash test data in a comprehensive way, while achieving a higher correlation between the KE HVMs and automotive prediction models. Additionally, this approach is able to consider the material characteristics of the tested UASs, their structural density and behavior during the impact. The assessment can be performed either directly with the corresponding impactor tests, or via relations of the criteria to the KE. Comparison against a defined, precisely verified and validated dataset could enable assessing the corresponding risk in a simpler way, based on the ATD response. Utilizing such a generalized procedure would enable the accumulation of a database of mutually compatible data. This may be seen as a challenging task, however, the current trend towards more detailed and accurate evaluation of the inherent risks of a particular UAS necessitates the use of more complex analysis.

The assessment of available data shows that proposed utilization of the energy transfer slope determined through peak head acceleration (Method C and D [4]) may not be suitable. The peak head acceleration shows higher variation than HIC and the assumed linear relation on KE, as shown in the case of the DJI Phantom, may not be applicable for all UASs. In the case of limited testing facility capabilities, when tests cannot be performed at the critical speed or terminal velocity, the HIC15 relation to KE seems to be more accurate than linear trend prediction. While the linear trend was suitable to describe the peak head acceleration relation to KE for a rigid impactor, the proposed methodology may lead to an overly restrictive prediction if applied to the more pliable and deformable structure of UASs, especially if the tests are not performed at the maximum impact speed. Unfortunately, due to limitations of available data, the relation between the peak head acceleration and KE could be performed only for the DJI Phantom drone series.

The limitation of the proposed approach is its high dependency on the repeatability and validity of performed tests. The position and orientation of the ATD, the location of the impact and other impact parameters are crucial for its applicability. Current standards do not ensure subsequent data compatibility due to the limited knowledge and introduction of completely new techniques for UAS assessment. Furthermore, this approach has only been validated and tested for a vertical impact to the top of the head. This could be a severe issue if the validation is not successful for other impact orientations or failure scenarios. Oblique impacts and impacts to other body regions are planned for testing in the coming years to extend and verify the applicability of the proposed approach. Therefore, there is a need for further research and data collection.

Due to necessity to relate the tests to previous impact studies performed with actual UASs, the test configuration with an ATD seated on a rigid chair and vertical impact was selected. Several studies have shown, that the ATD have a different response to the vertical impacts than a human. This leads to limitations for a direct application of the results to the real-world impacts. However, that was of the aims of the research, to use the ATD response, which may differ significantly, and relate it to the UASs impact tests and explain potential inconsistencies between criterions or the predictions of the transmitted energy. While the measured response for a rigid seating is higher than in case of free moving impact scenarios, such as standing or walking person, it means that the results are still conservative and do not introduce an unnecessary risk. Horizontal or angled impacts have proven to introduce higher loads and lead to a different response from the ATD, however, these were out of the limitations set for this study. The variability of the predicted injury probability also presents an issue. Automotive criteria can be simply utilized due to set threshold limits, however, transitioning to the predicted probability of AIS is not trivial.

Lastly, the proposed indirect estimation of the maximum transmitted energy showed a certain level of inconsistency at low impact kinetic energy. The impactor tests were conducted only for impacts in the range of 5–180 J. The variability of the data for low impact KE, caused by only minimal responses from the ATD, leads to differences in estimated transmitted energy. While the estimated transmitted energy varies based on the criterion used, a rational analysis may be used to select the most reasonable estimation. However, the peak head acceleration may lead to a prediction of higher transmitted energy than the impact KE. It is necessary to utilize the proposed approach only within the limits of the comparative impactor tests. Nevertheless, this should not affect its usability due to the necessity to perform assessments and risk estimations only for UAS with impact KE above 80 J.

Overall, the study shows that rigid impactor tests provide a reliable basis for estimating the kinetic energy transferred to the ATD during a drone impact. The results demonstrate clear and scalable relation between KE and key biomechanical criteria, while highlighting the significant influence of UAS deformability on energy transfer. The derived dataset allows for more consistent evaluation across different platforms and helps explain differences between KE-based vulnerability models and automotive HVMs. Although this approach is currently validated only for vertical impacts to the top of the head, the presented results form a solid basis for future extensions, including oblique impacts.
